# Motivational intensity and visual word search: Layout matters

**DOI:** 10.1371/journal.pone.0218926

**Published:** 2019-07-23

**Authors:** Marco Filetti, Oswald Barral, Giulio Jacucci, Niklas Ravaja

**Affiliations:** 1 Helsinki Institute for Information Technology HIIT, University of Helsinki, Helsinki, Finland; 2 Helsinki Institute for Information Technology HIIT, Aalto University, Helsinki, Finland; 3 Department of Information and Service Economy, School of Business, Aalto University, Helsinki, Finland; 4 Department of Social Research, University of Helsinki, Finland; University of Tübingen, GERMANY

## Abstract

Motivational intensity has been previously linked to information processing. In particular, it has been argued that affects which are high in motivational intensity tend to narrow cognitive scope. A similar effect has been attributed to negative affect, which has been linked to narrowing of cognitive scope. In this paper, we investigated how these phenomena manifest themselves during visual word search. We conducted three studies in which participants were instructed to perform word category identification. We manipulated motivational intensity by controlling reward expectations and affect via reward outcomes. Importantly, we altered visual search paradigms, assessing the effects of affective manipulations as modulated by information arrangement. We recorded multiple physiological signals (EEG, EDA, ECG and eye tracking) to assess whether motivational states can be predicted by physiology. Across the three studies, we found that high motivational intensity narrowed visual attentional scope by altering visual search strategies, especially when information was displayed sparsely. Instead, when information was vertically listed, approach-directed motivational intensity appeared to improve memory encoding. We also observed that physiology, in particular eye tracking, may be used to detect biases induced by motivational intensity, especially when information is sparsely organised.

## Introduction

Cognitive scope, defined as the attentional or mnemonic preference for central (dominant) or peripheral (secondary) details, has been linked to a number of affect-related factors such as mood [1, 2], anxiety (emotion) [3], hemispheric activity [4, 5], arousal (or activation) [6], valence [7–9] and motivational intensity [10]. Modulation of cognitive scope has also been attributed to individual traits, such as anxiety [11] or hemispheric dominance [4, 12]. Cognitive scope is sometimes described as the attentional or mnemonic preference toward the “trees” (narrow scope) or the “forest” (broad scope). Note that there may be some overlap between the affective phenomena which modulate cognitive scope. Emotions can be decomposed into three dimensions: arousal (also known as activation), valence (whether the emotion is positive, such as happiness, or negative, such as sadness) and dominance (whether it is characterised by domination or submission) [13]. Manipulating any of these components could cause cognitive scope to be altered. However, an additional component has been argued to be central to the modulation of cognitive scope. This is motivational intensity, defined as the driving force towards a stimulus (approach motivation) or away from it (withdrawal motivation) [14]. For example, anger is normally associated to slightly negative valence, high arousal and high motivational intensity. Hence, without controlling for confounding variables, the narrowing effect of anger could be attributed to high motivational intensity, high arousal or negative valence simultaneously. Continuing from this example, recent work which did account for confounding variables indicated that the high motivational intensity component of anger is indeed a major factor in cognitive scope reduction [15]. Anger is considered high in motivational intensity since it drives attention towards a specific goal (e.g. an enemy that needs to be attacked). On the other hand, affective states such as joy or relaxation are considered to be low in motivational intensity: these emotions do not drive towards or against a specific goal when being experienced (since the goal may have already been achieved). Such emotions have been linked to broad cognitive scope in experiments that employed Navon letters or Kimchi and Palmer stimuli [10, 14]. More in detail, high motivational intensity *indirectly* induced participants into paying more attention to details at the expense of general information. Note that we use the word *indirectly* as motivational intensity was induced using *task-irrelevant* manipulations, via ‘flanker’ tasks. This is important since it would not be of particular interest to observe an increase in interest towards a particular goal if a person is highly motivated in achieving that very goal. Instead, we are interested in the effect of motivational intensity on incidental or competing tasks. In this paper we focused on visual search, as we hypothesised that cognitive scope alterations would in turn affect visual search.

Apart from cognitive scope, motivational intensity has also been linked to increased cognitive control [16, 17]. That is, reward expectation may increase resource allocation in the brain, allowing the optimisation of behaviour and working memory capacity at the expense of other systems, resulting in increased physiological activation and strain. This effect is especially noticeable when utilising task-relevant manipulations [18]; during visual search tasks, participants quickly adapt to the task at hand, optimising their search strategy [19]. Importantly, increased cognitive control has also been observed during task-irrelevant manipulations [16, 20].

As previously mentioned, broadening and narrowing of cognitive scope can also be modulated by valence. That is, positive emotions (happiness) are said to broaden scope for attention and memory, while negative emotions (sadness) tend to narrow it [7–9, 21, 22]. It has been argued that this is because positive moods enhance the currently most accessible thought. Positive emotions are believed to broaden cognitive scope since the “default” (most accessible under neutral circumstances) scope is broad [23]. That is, the normal approach to a situation would be to consider it as a whole, rather than focusing on the details. Positive emotions, then, encourage this behaviour. On the other hand, negative emotions counter this “default” broad scope, narrowing it instead [24]. It is particularly relevant to our context that approach-motivated positive affect has been shown to improve recall rates for centrally presented words in a task-irrelevant manipulation [20].

In this paper, we assessed whether motivational intensity and valence influence performance during a common computer-based task: visual search. We carried out our investigations by manipulating both motivational intensity and valence directly, without employing specific emotions (i.e. we did not attempt to elicit anger or sadness). As in the previously cited work, we employed a ‘flanker’ task; this enabled us to investigate the effects of manipulations on visual search performance without directly influencing it. In other words, it would not be surprising for us to observe an increase in visual search performance if participants were motivated in searching faster or more accurately (assuming no additional controlling variables). Instead, we attempted to alter cognitive scope, expecting that this would in turn influence performance.

Factors modulating cognitive scope are relevant to the implementation of human-computer interfaces. This is because some systems present large amounts of scattered information, such as dynamic control applications (e.g. air or naval control systems). Given the complexity of the information presented, some may be overlooked if the current user of the system pays attention only to a limited interface area. It would therefore be valuable to detect such biases in order to reduce risk of failures due to human error (e.g. raise a low attention exception, which could, for example, alert the current user or other users of the system). This observation applies to systems beyond dynamic control applications: emerging search engines are starting to utilise result layouts which forego the currently ubiquitous list format. Instead, search results are sometimes displayed across larger areas, plotted in two dimensions, in a fashion that resembles scatter plots. This is done in order to provide personalised layouts [25], visually build queries [26] and/or refine queries over multiple iterations [27–29]. Apart from search engines, information obtained from web pages can be summarised into tag clouds, arranging keywords across a two-dimensional space [30, 31]. It would therefore be of interest to investigate whether users who rely on information scattered across a two-dimensional space are susceptible to attentional and mnemonic biases induced by high motivational intensity or negative affect.

We are also interested in real-time detection of such biases. Psychophysiological signals can be utilised to estimate the user’s emotional state [32, 33]. Systems that utilise psychophysiological signals have been able to automatically estimate stress, anxiety or mental workload [34–36]. We are interested in investigating whether multi-modal psychophysiological systems could also be used to estimate motivational intensity (and, in turn, cognitive scope). Motivational state estimated via psychophysiology could help in preventing or detecting the aforementioned biases during the use of systems which depend upon the users’ visual attention.

In particular, eye tracking could be very informative for the detection of changes in the users’ cognitive scope. It has been previously indicated that less attention is paid to the periphery of a screen when motivational intensity is heightened [20]. Moreover, it has been shown that participants adapt eye movement strategies to optimise performance in various visual search tasks [37]. Given that motivation may guide what the visual system presents to conscious awareness [38], it is reasonable to expect that the effects of motivational manipulations would be reflected in the users’ search strategies and would be measurable via gaze pattern analysis.

These are additional signals that may also be helpful in the detection of cognitive biases. Motivational intensity is normally associated to increased sympathetic nervous system activation (arousal) [39]. This is associated to increases in heart rate [40] and skin conductance [41]. Motivational intensity has also been linked to EEG asymmetry: activity in the left hemisphere raises as motivational intensity increases [12, 42–44]. Moreover, left hemispheric activity has been associated to a localised cognitive scope, while grater right (or lower left) activity is correlated a broader scope [5, 45, 46]. This suggests that changes in motivational intensity could be detected by monitoring a number of relatively simple signals: Heart Rate (HR), Electrodermal Activity (EDA) and EEG (Frontal Alpha Asymmetry, which can be measured using only two electrodes). Using signals that require low computational power renders them appropriate for real-time usage (also known as on-line analysis).

In summary, the picture that can be drawn from the literature is that motivational intensity modulates attentional scope. Changes in scope may affect users which are required to monitor relatively large amounts of information at a fast pace. In this context, it is necessary to take into account the effect of task-irrelevant stimuli, which reduce attentional resources allocated to the main task. It is also possible that the same information displayed in different ways would be not affected by motivational biases in the same fashion. For example, attentional biases that narrow cognitive scope may decrease performance more noticeably when the user of the system is parsing information displayed sparsely. Eye tracking and psychophysiological signals could be used to detect such motivational biases in real time. Finally, valence can also affect cognitive scope: it is often narrowed by negative affect.

### Studies

We present three studies which we conducted to assess the effect of motivational intensity on visual word search, while simultaneously testing whether any biases induced by it can be detected via multiple psychophysiological signals. This is something that, to our knowledge, has not been previously investigated.

The first study assessed whether motivational intensity does affect visual search differently depending on the way in which information is presented. Studies 2 and 3 were follow-up studies that we conducted after interpreting the effects we observed in the first study. Pre-empting our findings, in the first study we observed that lists and scattered information were being affected differently by our manipulations. We then conducted two additional studies to investigate this finding in more detail, one solely focused on lists and the other on scatters. This approach allowed us to investigate to what extent the effects of the cognitive biases we induced were dependent on the visual layout employed.

The trial design was inspired by a previous experiment that demonstrated that visual attention allocated to peripheral areas of a computer screen is reduced when motivational intensity is high [20]. We built our hypotheses on this observation, adding a number of important considerations to our experimental design. In our studies, we considered three different motivational state conditions: neutral, approach and withdrawal. Across the studies, we explicitly asked participants to perform visual searches, while altering visual presentation layout at every trial. Our trial design included a questionnaire phase and the end of each trial, providing more granular data. This allowed us to determine whether cognitive scope manipulations affected active visual searches, rather than incidental information. Moreover, we included psychophysiological signal collection and analysis, in order to assess these signals could be used for attentional bias detection, particularly in real-time systems.

Our approach sits in between basic and applied research. From a basic research perspective, we measured the effect of motivational intensity on visual search. From an applied research perspective, we investigated whether attentional biases can be detected via psychophysiology, and if these biases are equally present when visual presentation layout is altered. We believe there is a value in bridging basic and applied research: often, basic research does not provide enough information to investigate practical problems, while purely practical, on-site studies run with little experimental control do not provide enough detail for the investigation of the underlying cognitive phenomena [47].

### Hypotheses

The three studies we ran assessed three common hypotheses. The first hypothesis assessed whether motivational intensity does affect visual search, and whether different visual (or semantic) information organisation approaches are affected differentially when motivational intensity is high. The second hypothesis explored whether motivational intensity can be estimated via psychophysiological signals. Note that the second hypothesis follows from the first. That is, estimating motivational state via psychophysiology is a valuable effort if it can be used to detect attentional biases. The third hypothesis is secondary; it assessed whether affective state affects memory (it was tested after the visual search tasks were completed).

The three studies had a common structure: they were composed of a relatively large (> 100) number of trials. Each trial started with a word search task, which consisted in category identification. This was followed by a reward expectation phase, inducing three different levels of motivation: neutral, approach-directed and withdrawal-directed. Several words were then displayed on a computer monitor using different layouts, depending on the study (e.g. listed, scattered, coloured or clustered). During this phase, the aforementioned physiological signals were recorded: these were electroencephalography (EEG), electrodermal activity (EDA), electrocardiogram (ECG) and electro-oculogram (the latter only used to assist in EEG artefact rejection). We used EDA and ECG to measure activation and EEG to assess whether increased motivation would result in frontal asymmetry. Eye tracking was employed to monitor changes in visual search strategy induced by motivational manipulations. Word presentation was followed by the ‘flanker’ task, after which affective manipulations were implemented via reward feedback, which could be positive, negative, or neutral. Participants performance in the visual search task was assessed after our motivational and affective manipulations.

Our hypotheses are briefly outlined below. They will be reiterated in more detail after introducing the implementation of the first study—which acted as a template for the remaining two studies—in the “Hypothesis” section.

#### Hypothesis 1: Motivational intensity modulates cognitive scope and interacts with search paradigm

The first hypothesis is focused on the effect of motivational intensity on attentional and mnemonic scope and how it interacts with visual search tasks.

We elicited high motivational intensity in two directions: approach and withdrawal (also called avoidance). It is important to assess both directions when exploring motivation-related phenomena on human behaviour [48]. These two conditions were compared agains a ‘neutral’ baseline, representing low motivational intensity. We expected to find a main effect of high motivational intensity on performance, so that as it increases, performance decreases. This is because a narrow scope would result in lowered attentional allocation to words presented in peripheral areas [20].

A main effect of motivational intensity on eye tracking metrics correlated to attentional scope would also support this hypothesis. For example, the amount of time spent looking at individual words should increase when motivational intensity is high, while the average distance of gaze from the centre of the screen should decrease (to reflect a narrow attentional scope). In other words, increased motivational intensity would induce participants in spending more time observing the “trees” rather than the “forest” [15]. This is based on the observation that high motivational intensity is correlated to increased attention towards a central area [20]. In our case, however, the locus of attention is relative, rather than absolute (e.g. attention is centred around a cluster of words, rather than the centre of a computer monitor).

Central and novel to the studies presented in this paper is the potential interaction that might arise in how search is performed and the level of motivational intensity. For example, when information is presented in a sparse fashion (e.g. scattered), one could expect to find an increased adverse effect of high motivational intensity. On the other hand, when words are presented densely (e.g. listed) high motivation may be less detrimental (or even beneficial), as a narrow scope would focus attention on a specific area of the screen, populated with words. In Studies 2 and 3 we altered semantic density (rather than visual density) as words were always visualised in the same way.

#### Hypothesis 2: Physiology is influenced by motivational intensity and predicts errors

**Hypothesis 2a**. We expected our motivational manipulations to affect physiology measured during the word search task.

We measured EEG Frontal Alpha Asymmetry (FAA); usage of this metric is advantageous as it requires little computational time and could be employed in real-time systems to detect (or prevent) attentional biases. We also measured inter-beat interval (or IBI, via ECG) and electrodermal activity (EDA, or skin conductance).

We expected to find a main effect of motivational intensity on three variables: EEG asymmetry was expected to increase after reward expectancy manipulations. Specifically, we expected relative left activity to increase along with motivational intensity [46, 49]. This is observable as an increase in right alpha band power. We also expected increases in heart rate and in skin conductance variables, indicating sympathetic nervous system activation [40, 50, 51]. Therefore, we expected IBI and EDA to increase along with motivational intensity.

**Hypothesis 2b**. We also assessed the possibility of predicting performance via the aforementioned physiological signals, with the addition of eye tracking. Given the exploratory nature of this test, we tested all variables simultaneously, for all studies, correcting for multiple comparisons. This is because we could not precisely determine which signals were most likely to predict performance; instead, we speculated that signals could predict performance in any direction. For example, assuming that high motivation reduces scope, we expected high sympathetic nervous system activation to be correlated to lower performance. Similarly, we expected performance to decrease as the distance of the eyes from the centre of the screen decreases, as measured by eye tracking. EEG (as measured by alpha band power) was also expected to correlate with performance, as, in turn, we expected it to be correlated to motivational intensity. However, as previously indicated, we could not predict which of these signals would correlate with performance, and in which direction. Given that we corrected for multiple comparisons, eventual significant findings would indicate which signal could be suitable for automatic detection of biases induced by motivational intensity during visual search.

#### Hypothesis 3: Affect modulates memory and interacts with search paradigm

In addition to reward expectancy, we manipulated reward feedback to induce positive or negative affect, in varying levels. Similarly to hypothesis 1, we tested whether this had a main effect on performance and whether it interacted with search paradigm.

Rewards were communicated to participants after the word presentation but before assessing performance. Therefore, we could only test for mnemonic biases, as our manipulation took place after participants performed the search task.

We expected to find a main effect of reward feedback on performance. Negative affect has been demonstrated to increase localised attention, as does high motivational intensity [3, 10]. Hence, negative rewards were expected to induce narrower scope, decreasing the number of words remembered. Similarly to Hypothesis 1, we expected an interaction between reward feedback and search paradigm, especially considering that the centre of attention may not always correspond to the absolute centre of the screen. For example, sparse layouts may be more adversely affected by narrower mnemonic biases induced by negative affect.

### Study design

Design and methodology common to all three studies are outlined in this section. The sections Study 1 to Study 3 will describe the trial structure and detail how each study differed from the previous.

Our studies were composed of a sequence of trials (180 trials in Study 1, 108 in studies 2 and 3). Each trial comprised 6 phases. These were:

1. Category assignment: participants were given one (in Study 1) or more (studies 2 and 3) target categories. They were instructed to memorise all words belonging to the given target categories.
2. Expectancy cue: one out of three possible cues were shown to participants. These were *Neutral* (the next trial will not be rewarded), *Approach* (you may gain a reward in the next trial) or *Withdrawal* (you may lose money in the next trial). This 3-level variable is called *Cue*. It was balanced within participants (one third of the trials were preceded by each queue, in randomised order).
3. Word presentation: Participants were instructed to find words belonging to the target categories. Although participants were not rewarded for this task, they were instructed to perform it as best as they could. Words were presented to participants in various ways, specific to each study. Words were presented using differing visual arrangements in Study 1; this variable was called *Layout*. In Studies 2 and 3, the amount of categories presented simultaneously was manipulated; this variable was called *Diversity*. These variables were also balanced within participants, and fully crossed with *Cue*.
4. Flanker: participants were instructed to press the left or right key on the keyboard as quickly as possible, depending on the direction of a symbol shown on screen. They were told that they would be rewarded according to their speed in responding correctly to this task. The flanker task allowed us to manipulate motivational intensity on a trial-by-trial basis, independently of the *Cue*, *Layout* (Study 1) or *Diversity* (Studies 2 and 3) variables.
5. Reward feedback: participants were awarded a small amount of money (in half *Approach* trials), or lost a small amount of money (in half *Withdrawal* trials) or neither. Rewards were balanced within participants and across conditions. They did not depend on reaction times (with some exceptions, see the “Reward feedback” section). This variable is called *Feedback*.
6. Word recognition: A list of words belonging to the target category(/ies) was shown. Half the words did appear during word presentation (phase 3), while the other half was used to measure false alarms. We calculated the number of *Errors* participants committed in this phase to assess the effect of the independent variables mentioned above.

#### Word creation

We manually created a dataset of 120 words for Study 1 and 168 words for Studies 2 and 3. In Study 1, the 120 words were grouped in 12 categories containing 10 words each. In Studies 2 and 3, the 168 words were assigned to 14 categories, each containing 12 words. The categories were created in order to be non-overlapping, while the individual words were selected to be non-salient; for this reason, we excluded particularly long, short or otherwise peculiar words. Emotional words were excluded by verifying their rating in the affective norms database in Finnish [52], or if a Finnish rating was not available, using the corresponding English translation [53].

#### Stimulus presentation

All stimuli were presented on a 22” widescreen LCD monitor, with a refresh rate of 59 Hz and resolution of 1680 × 1050 pixels. Participants were placed at a distance of approximately 60 cm from the monitor; the whole screen resulted in a rectangle covering a visual angle of approximately 42.78° × 28.07°. Stimuli were presented using PsychoPy version 1.81 [54]. Words were formed by Lucida Console characters of 20 in height, corresponding to a visual angle of 0.76°. Screen background was white and words were black (except when displayed within a condition called “colours”, present only in Study 1).

#### Recording apparatus

Data were recorded using a Brain Products QuickAmp recorder (BrainProducts, Munich, Germany). We bandpass filtered data at recording, with a high-pass of 0.01 Hz and notch filtered at 50 Hz. We recorded Electroencephalographic data from 32 electrodes, arranged using the 10-20 standard (Jasper, 1958). We recorded electro-oculograms from the left and right eyes using two bipolar electrodes (HEOG and VEOG). During recording, we used the average of all channels as reference (common reference). EEG impedances were at most 15 kΩ (acceptable for this type of research [44, 55–58]). Note that the average impedance recorded for the channels used in the analysis was noticeably lower (*M*: 3.83 kΩ, *SD*: 3.41). ECG was recorded using a bipolar lead compatible with the QuickAmp recorder. EDA was recorded using an adaptor compatible with QuickAmp auxiliary channels. Gaze position was tracked by a RED500 eye tracker (SensoMotoric Instruments, Teltow, Germany). Timing markers were sent from the stimulus presentation computer to the eye tracking computer and EEG amplifier simultaneously using a three way (‘Y’) parallel cable, with a crossover (LapLink) adaptor attached to the eye tracking computer. Signal processing procedures are detailed in the “Measures” section.

### Participants

All participants were students or staff at Aalto University or Helsinki University. The study was announced using the respective universities’ advertising services. In the invitation, prospective participants were told that they would be paid a maximum of 70 € and a minimum of 20 €, depending on how their reaction times compared to other participants. All participants were paid 70 € at the end of the experiment. Since rewards were balanced across conditions and did not depend on reaction times, some exceptions were put in place to avoid this deception from being noticed (described in the “Reward feedback” section). Immediately after the experiment but before being paid, participants were asked whether they believed their rewards were being allocated fairly. Participants who replied that rewards were not fair were paid but excluded from further analysis. Across the three studies, two participants were excluded for this reason (one in Study 1 and one in Study 2).

All participants were native Finnish speakers, right handed, non colour-blind, with otherwise normal or corrected-to-normal vision.

### Ethics

Study design and recruitment procedures were approved by the Aalto University Ethical committee. Approval numbers 2014_18 (Study 1) and 2016_02 (Studies 2 and 3). Written consent allowing the analysis of raw data and the publication of averaged results was obtained from each participant.

### Measures

As mentioned in the introduction, we employed measuring techniques that could theoretically be implemented in a real time system and hence required a relatively low number of sensors and modest computational power. These were 2-electrode EEG asymmetry, 3-sensor ECG (for IBI), 2-sensor EDA and eye tracking. We employed 32-channel EEG at recording to perform ICA (in order to decrease the risk of Type I errors in the subsequent analyses).

This section describes how data were analysed across all three studies. Each describes how we calculated the variables we utilised for our statistical tests. Each variable is written in italics.

#### Data analysis

For all data segments, we considered physiological activity recorded only during the word presentation phase. This was done to exclude noise caused by muscular activity from analysis, since participants were instructed to stay as still as possible during this phase but not in the other phases (also considering that the following phase contained the flanker task, which required rapid body movements).

In addition to the previously mentioned signals, we measured electro-oculogram (EOG) to reject EEG trials potentially containing eye blink artefacts. Specific rejection criteria for each signal are defined in the following subsections, while the number of participants that contributed to each measurement (indicated by *N*) is reported separately in the “Results” subsection of each study, along with their respective statistical test results.

#### EEG analysis

During analysis (i.e. after recording), EEG data were filtered with a high-pass of 0.5 Hz and low-pass of 70 Hz and re-referenced to common average. Ocular artefacts were removed by performing an Independent Component Analysis (ICA) back-transform in Brain Products Analyzer 2: components which were deemed to represent vertical and horizontal eye movements (typically the first two) were removed after visual inspection. EEG data were rejected if EOG channels appeared to contain an excessive amount of noise during visual inspection.

After recording, the data segment of interest (word presentation phase) was split into three segments of 1 s each. Segments were rejected if the scalp electrodes contained activity exceeding ± 200 μV (± 400 μV for EOG), in line with previous research utilising a QuickAmp amplifier [59–62]. The median number of EEG trials rejected per participants were 5 in Study 1 (*M*: 11.3, *SD*: 24), 2 in Study 2 (*M*: 6.2, *SD*: 9) and 5 in Study 3 (*M*: 16.9, *SD*: 30). As in previous research [63], we computed a frontal asymmetry index on midfrontal electrodes (F3 and F4), subtracting the natural log of alpha power at the left electrode from the natural log of alpha power at the right electrode. Alpha band was defined as the average between 8 Hz–12 Hz. Power was computed using Welch’s method [64] in Matlab (pwelch), with a window size of 75% and 50% overlap. This procedure was repeated for each valid segment of EEG data in every trial. The average of the segments was taken as an asymmetry index for the given trial, with higher scores indicating greater left activity. As in our previous research [44, 58], we utilised resting states between trials for baselining. This is because a single baseline measurement would not be sufficient to account for overall variability across the experiment [63]. That is, the asymmetry index for a given trial was computed by subtracting the index measured during the word presentation to the value measured 1 s before the start of trial itself (this time window corresponded to inter-trial blanks, which took place after the questionnaire phase of the preceding trial ended). We called this variable *FAA*.

#### ECG analysis

ECG data were analysed to measure activation induced by motivational intensity manipulations (i.e. by *Cues*). ECG data were rejected on a participant-by-participant basis after visual inspection, before analysis. For every 3 s segment (starting from stimulus onset), we first de-trended data by subtracting a fifth-degree polynomial fitted via least squares. Time series data were then full-wave rectified, removing polarity. We Searched for peaks in Matlab, using the ‘findpeaks’ function with a minimum peak distance parameter of 200 ms and a minimum peak height parameter of 50% of the global maximum found within the given segment. All peaks found via this method pinpointed a QRS complex in time (specifically, R peaks). Inter-beat intervals (in ms) were calculated as the average distance between the found peaks in the given segment. We call this variable *IBI*.

#### EDA analysis

As for ECG, EDA data were analysed during the word presentation phase and were rejected on a participant-by-participant basis after visual inspection. EDA data were decomposed into phasic and tonic signals using Ledalab [41]. Given that average phasic activity (as computed by Ledalab) is mostly contained within a 1 s–4 s window from stimulus onset, Integrated Skin Conductance Responses (*ISCR*, in microsiemens, or μS) were calculated by summating phasic activity from 1 s to 4.0 s after the beginning of the motivational manipulation. Dividing the obtained summation by the number of samples per second in each segment resulted in a μS/s (microsiemens per second) measurement for each trial. This variable is called *ISCR*.

#### Eye tracking analysis

We calculated three eye tracking variables per trial. These were 1) *Gaze duration* (total dwell time on word) 2) *Words skipped* (words not seen per trial) and 3) *Radius* (average distance of on-screen gaze position from the screen’s centre).

There were 2 conditions that had to be satisfied in order to mark a word as ‘seen’. 1) Given that the human eye can identify words within approximately 3 (horizontally) and 5 (vertically) degrees of visual angle [65], the participant’s estimated gaze had to fall within a rectangle surrounding the given word, measuring approximately 5.53° × 3.83° of visual angle (corresponding to 5.8 cm by 4 cm, or 200 by 150 pixels). 2) The estimated gaze had to stay within an inner square covering 2.76° of visual angle (corresponding to a side of 2.9 cm, or 100 pixels) for at least 50 ms; this indicated a fixation [66]. The total time spent within the inner rectangle defined our *Gaze duration* metric.

At the trial level, we calculated the mean distance of gaze from the centre of the screen by applying Pythagoras’ theorem to eye tracking coordinates; this variable was called *Radius*. Eye tracking data were rejected at the participant level when calibration accuracy under 1° could not be achieved. Moreover, data from individual trials were rejected if no eye movements were detected for least 80% of the trial duration. Data points indicating positions of 0, 0 (exactly the middle of the screen) were considered missing, as SMI eye trackers use this value to indicate data absence.

#### Performance

For each trial, we measured performance by calculating the overall number of *Errors*, based on the answers given in the recognition phase of the given trial. *Errors* were defined as the number of false alarms (words belonging to the target category, but not actually presented during the trial) plus misses (shown in the given trial, but not reported as seen by the participant).

#### Statistical analysis

We organised our data in two tables: one containing trial-level data (ECG, EDA, EEG, number of words skipped, mean gaze distance from centre of the screen, errors, etc.) and one containing eye-tracking word level data (e.g. gaze duration for that word, whether word was seen, or remembered). As previously mentioned in the “Study Design” section, all our tests were within-participants: each participant performed the task under all conditions we tested.

Our hypotheses (reiterated later in each study-specific “Hypotheses” section) were tested by fitting a generalised mixed model to the data. The hypotheses were formulated a priori by examining the literature previously presented in this paper. We reported all results, whether statistically significant or not. Bonferroni correction was applied to all p-values related to the same hypothesis, within the same study.

We applied two-level testing. Firstly, we fitted a linear model in Matlab using the ‘fitglme’ function. Secondly, we performed an ANOVA on the resulting model (in Matlab: lmg = fitglme(…); anova(lmg)). Thirdly, only if the top-level ANOVA test was significant at a Bonferroni corrected alpha of .05, we reported the individual factor effects resulting from the first test. The individual effects p-values were also Bonferroni corrected.

We corrected for 5 comparisons when testing Hypothesis 1 in Study 1 (1 test on *Errors* + 3 tests on eye tracking variables + 1 test on *Outlier hits*) 4 comparisons when testing Hypothesis 1 in Studies 2 and 3 (which did not include *Outlier hits*), 3 comparisons for the correlation between motivational intensity and physiology of Hypothesis 2a (*ISCR*, *IBI* and *FAA*), 12 comparisons for the exploratory prediction of errors via physiology in Hypothesis 2b and 1 comparison in Hypothesis 3 (no correction, since we only performed one test on *Feedback*).

Response variable distribution was specified as the type of distribution that most closely represented the probability density of our response variable. We selected the Poisson distribution for *Errors* and *Words skipped*. The binomial distribution was selected for *Outlier hits* (specific to Study 1, see the “Eord presentation” section). Non-normally distributed variables (*IBI* and *ISCR*) were log-normalised. Predictors were specified as fixed effects and participant numbers as random effects. Using this method, we calculated t-statistics and p-values.

Means and standard deviations were calculated separately for each test. Effect sizes were calculated using r_equivalent_, given that no standardised method has yet been accepted for use on mixed models [67]. Similarly to means and standard deviations, r_equivalent_ scores were calculated separately for each statistical test we conducted [68], using the Measures of Effect Size (MES) toolbox for Matlab [69].

### Additional analyses

The analyses discussed in this section were not devised as part of our original design. Instead, their inclusion was suggested by the reviewers of the present article in order to support its findings. The results of these analyses will be reported in the “Additional analyses” section, along with the summarised results of the three studies.

#### Parietal alpha

Parietal EEG alpha power is considered to be inversely correlated to cognitive and memory load [70–72]. We measured alpha power for two reasons. Firstly, we were interested whether load was higher during the word search phase, when compared to baseline. Secondly, to assess the effect of *Diversity* (a variable present only in Studies 2 and 3).

We measured alpha power using the same parameters we used for asymmetry (Welch’s method, 1 second time windows, power between 8 and 12 Hz) except that we only considered the Pz electrode. To assess the overall effect of the word search task, we compared trial-level baseline alpha power indices against the middle second of the word search task indices in a paired t-test. The test was performed within participants (e.g. in Study 1 a single test involved 180 pairs, assuming no rejected trials). We repeated the test for each participant, counting the number tests that were significant at alpha .05. The tests were one-tailed, with the alternative hypothesis being that baseline alpha power would be greater than word search task alpha power (against the null hypothesis that their means were equal). To assess parietal alpha *Diversity*, we utilised a generalised model test (the same method we used to assess asymmetry for *Cue*) on Pz alpha power when compared to baseline.

#### Spill-over effect

We run this test to measure the effect of reward feedback on the following trial, rather than the trial during which it was presented. The test parameters were otherwise the same we used for our main reward feedback hypothesis (Hypothesis 3). That is, we tested the effect of *Feedback* on *Errors* committed in the following trial using a generalised linear model (resulting in 179 tests for participant in Study 1, for example).

#### Eye-tracking calibration

We also performed a test to assess whether the fact that we performed only one calibration session at the start of each experimental session had a detrimental effect on eye tracking accuracy. We measured the average distance between gaze position and the closest word shown on screen, for every trial. Similarly to our other tests, we utilised a generalised linear model to assess the effect of the trial number on the distance of gaze from the closest word.

## Study 1

We assessed the effect of high motivational intensity in opposing directions (i.e. approach and withdrawal) against low motivational intensity, using three reward expectancy cues (called *Approach*, *Withdrawal* and *Neutral*, respectively). Moreover, given that reward outcomes have not been previously shown to greatly affect memory using three levels [20], in our studies we considered five reward levels. These were *Neutral*, *Gain*, *Loss*, *Nongain* and *Nonloss*. We employed five different visual search paradigms; these were called *Scatter*, *Clusters*, *Clusters + outlier*, *List*, *Colours*) and are described in more detail in the “Word presentation” section.

This first study can be described by a 3 (*Cue*) × 5 (*Layout*) × 3 (*Feedback*) fractional factorial design, in which the *Cue* and *Layout* factors were fully crossed (reward outcomes were not fully crossed as money could only be gained after *reward* cues and could only be lost after *punishment* expectancy cues).

As previously indicated in the “Studies” section, this design may resemble ‘Experiment 1’ described in [20], but presents a number of important differences. Firstly, our design considers motivational direction (approach versus withdrawal). Secondly, we included five (instead of two) visual presentation layouts. Thirdly, considered five (instead of two) possible reward outcomes. Fourthly, the implementation of our flanker task (used to manipulate motivational intensity) was more complex, as described in the “Flanker task” section. Fifthly, we included EEG, EDA, ECG and eye tracking in our experimental design. Sixthly, participants were explicitly instructed to perform a search. These important differences allow us to investigate interactions between motivational intensity, affect, visual search and physiology.

### Method

**Fig 1:**
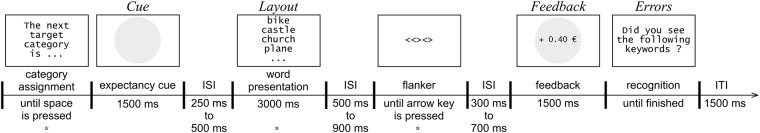
Trial structure, Study 1. Visual depiction of the trial structure described in the “Method” section. In this example, the circle was associated to a possible gain (*reward*) and a reward was eventually allocated. All trials followed this structure. Above each phase, the variable name of interest is shown in italics. Asterisks mark phases during which Study 1 differed from studies 2 and 3.

This experiment was composed of 180 trials (plus an additional 10 practice trials), split into four blocks by three breaks, one every 45 trials. Each break lasted as long as the participants desired. As previously stated in the “Study design” section, each trial comprised six phases: 1) Category assignment 2) Expectancy cue 3) Word presentation 4) Flanker 5) Reward feedback 6) Word recognition. This structure, along with the duration of each item and interstimulus / intertrial intervals is depicted in Fig 1. Each phase is described in more detail in the following subsections.

#### Category assignment

Each trial began with a category assignment screen, asking the participant to memorise words belonging to a specific category (e.g. “look for words belonging to the buildings category”). This category is called *target* in this paper, while any words that do not belong to this category are called *irrelevant*. These rules applied only to the current trial (participants were instructed about this during practice). Given that each cue / category combination was repeated 12 times, categories were paired so that every category could be selected exactly once as a target (and once as irrelevant) for every repetition. This procedure generated the 180 category assignments that were used in the 180 trials that composed the experiment (3 *Cue* × 5 *Layout* × 12 repetitions). The final trial ordering was shuffled, with the condition that every subsequent trial had to employ both different target and irrelevant categories (so that words belonging to the same category could not be displayed twice in adjacent trials).

In total, there were 12 possible categories (translated from Finnish, the original language of the experiment): buildings, vehicles, clothes, furniture, landscapes, chemical elements, fruits, body parts, animals, musical instruments, spices, stationery. Each category contained 10 words, all singular nouns of similar length.

#### Expectancy cues

Expectancy cues were presented as light grey (75% white) filled shapes contained within a square covering a visual angle of 6.68° (corresponding to a side of 70 mm or 100 pixels). The three cues were a triangle, a square and a circle. Each symbol indicated to the participant that in the current trial, a reward may be given if response to the flanker was faster than the average response of all participants (*Approach*), a negative reward may be given after a slower response (*Withdrawal*), or no reward would be given (*Neutral*). As previously indicated, rewards were eventually balanced and did not depend on participants’ reaction times (given some exceptions listed in the “Reward feedback” section). The meaning of each symbol (triangle, square and circle) was counterbalanced across participants. Ten practice trials allowed participants to familiarise with the meaning of each symbol. The three expectancy cues were evenly split across the 180 trials, in randomised order.

#### Word presentation

**Fig 2:**
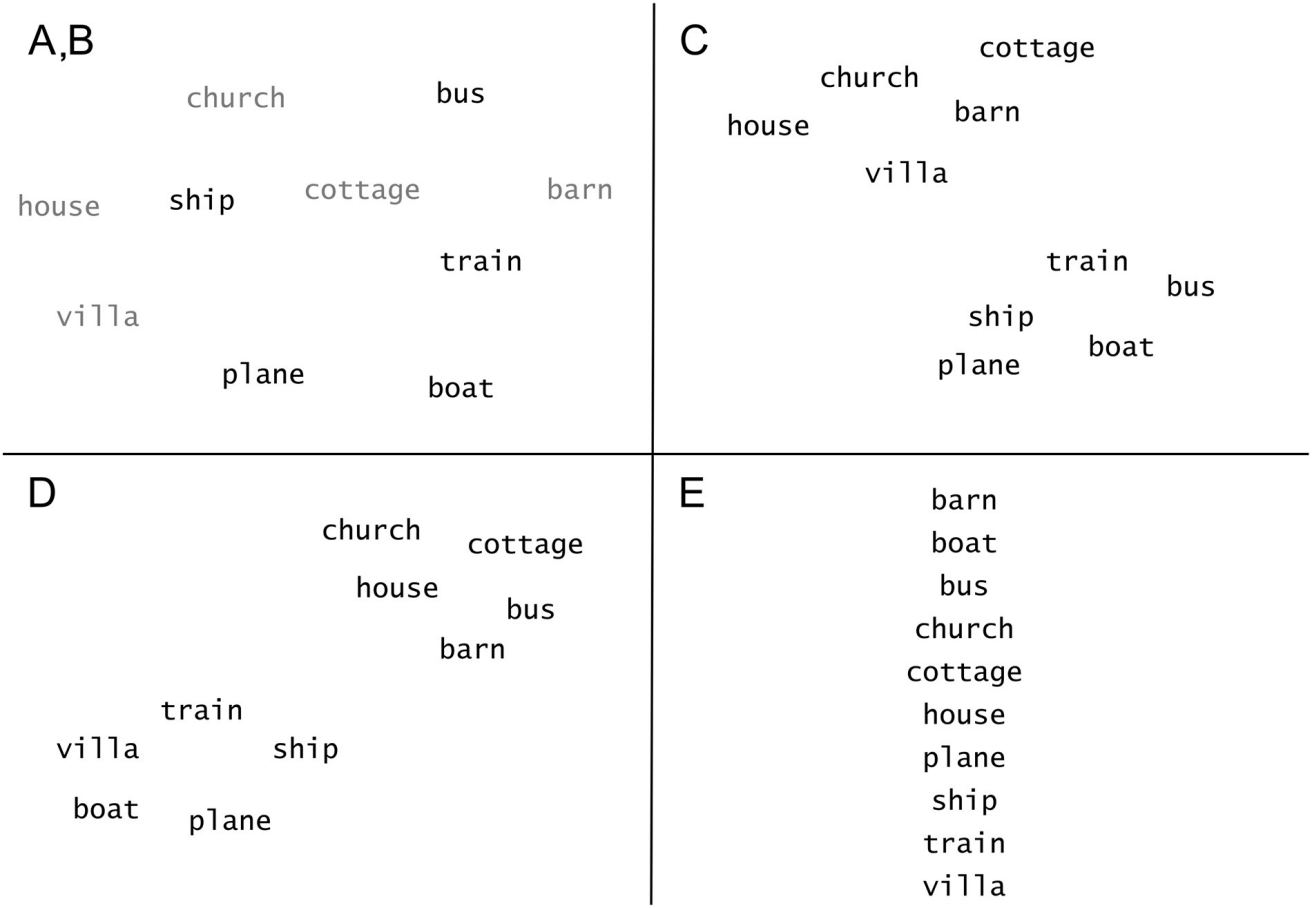
Word arrangements, Study 1. Example of word presentation layouts (conditions). **A**: Scatter and **B**: Colours (apart from using colours, both conditions shared exactly the same parameters). **C**: Clusters. **D**: Clusters + outlier. **E**: List.

We organised information in either concentrated or scattered approaches, and with or without visual category identification. We call this variable *Layout*. The five possible layouts (displayed in Fig 2) were:

- *Scatter*: words were pseudorandomly scattered across the screen (sparse layout without category identification).
- *Clusters*: words were visually clustered according to their category (concentrated layout with category identification). This was done by drawing an invisible ellipse that encircled approximately half the screen. Words from each category were placed around two antipodal points.
- *Clusters + outlier*: similar to clusters but with an outlier word (concentrated layout with category identification). One word, called the outlier, was swapped with another so that a target word appeared in the irrelevant cluster. This condition was introduced to examine the effect of motivational intensity on attention for peripheral details (in this case, the outlier word).
- *Colours*: words were pseudorandomly scattered across the screen, colour-coded according to their category (sparse layout with category identification). The two possible colours (assigned at random) were: dark blue and dark green (their RGB 0-255 codes were respectively 31,120,180 and 51,160,44). These colours were the two darkest colours generated by ColorBrewer 2.0 when requesting a colour set for four paired, colourblind-safe, qualitatively separated data classes [73]. This condition was used as an alternative method to encode category affiliation, as opposed to the previously described visual clusters. Colour has been shown to greatly influence efficacy of visual search when compared to other ways to alter the graphical appearance—eg luminance or chroma [74].
- *List*: words were aligned so that they were horizontally centred and equally distributed vertically (concentrated layout without category identification). Positions were arranged in ascending alphabetical order. This condition was included as ordered lists are commonly used in visual search tasks.

Care was taken to avoid particularly salient words. In all conditions (except *list*, in which their vertical position was equally distributed across the screen) words were separated by approximately 86° of visual angle (corresponding to 90 mm or 128 pixels).

#### Flanker task

After word presentation, the flanker stimulus was shown to participants. It was composed of five ‘<’ and ‘>’ characters, with the direction of each selected at random. Participants were told to press the left or right keyboard arrow corresponding to the direction of the middle character as quickly as possible so that they could be rewarded (after seeing the *Approach* cue) or not punished (after seeing the *Withdrawal* cue). The flanker was displayed at the centre of the screen.

To verify that our motivational manipulations were effective, we performed two paired t-tests on a participant-by-participant basis. We tested reaction times to the flanker after *Neutral* cues were shown against *Approach* and *Withdrawal*. The tests were one-tailed (the alternative hypothesis was that reaction times after *Approach* and *Withdrawal* were less, or faster, than after *Neutral*). The null hypothesis was rejected in all cases (80/80), indicating that our manipulations were indeed effective.

#### Reward feedback

After the flanker, participants received reward feedback. They could either gain 0.40 € (if fast enough, after *Approach* cues), lose 0.40 € (if slow, after *Withdrawal* cues) or neither. Rewards were pseudorandomly balanced so that half *Approach* trials would result in money being received and half *Withdrawal* trials would result in money being lost, the remaining half receiving no reward. All *Neutral* trials were followed by *Neutral* feedback (no money lost nor gained).

Some exceptions were put in place: during initial pilot testing, most participants noticed that rewards were being manipulated. For this reason, we made sure to reward very fast responses and punish very slow responses. Similarly, pressing the wrong arrow key always resulted in negative rewards. We defined the reaction time threshold as 1.5 standard deviations of the current participant’s reaction time distribution. We implemented these exceptions so that rewards were balanced (e.g. allocating a *Gain* because of a very fast reaction would result in a subsequent *Gain* trial being replaced with a *Nongain*). This ensured that very few participants noticed the reward manipulation (two across all studies). At the end of the experiment, participants were asked if they noticed anything “strange” about reward allocation. Participants who responded yes were discarded. We also intended to reject any participant who failed to reach 90% accuracy in flanker response, but in practice this threshold was never passed.

In total, the *Feedback* variable comprised five levels: *Neutral* (no reward when no reward was possible), *Gain* (positive reward in *Approach* trials), *Nongain* (negative reward in *Approach* trials), *Nonloss* (positive reward in *Withdrawal* trials) and *Loss* (negative reward in *Withdrawal* trials). This ensured that rewards were coded according to expectations, rather than outcome alone, as suggested by [75].

#### Word recognition

At the end of each trial, participants indicated which words they saw during the preceding word presentation phase. Ten words belonging to the target category (5 shown and 5 not shown) were displayed in a randomised list at the centre of the screen. Participants responded “yes” or “no” for each word by using the left and right arrow keys. Participants were allowed to make corrections to their responses by pressing the up and down arrow keys and re-entering a response until the end of the questionnaire phase (which ended 1 second after their last answer). This was the last phase for each trial, and was used to measure performance (i.e. *Errors*) for the given trial.

#### Participants

Data were collected from 40 participants (20 female). The number of participants is in line with our previous research employing within-participants design [44, 58, 76]. Their age ranged between 47 and 19 years (*M*: 25.70, *SD*: 6.27). An additional participant (whose data were discarded) noticed that rewards were not being awarded fairly.

### Hypotheses

#### Hypothesis 1: Motivational intensity modulates cognitive scope and interacts with search paradigm

We expected to find a main effect of *Cue* on *Errors*. This factor (*Cue*) had three levels, specified as nominal in our linear mixed model: *Neutral*, *Approach* and *Withdrawal*. We expected *Approach* and *Withdrawal* cues to decrease performance. *Layout* was coded as a covariate in this analysis, since category identification was easier under the *Colours*, *Clusters* and *Clusters + outlier* layouts.

We also expected to find a decreased number of *Outlier hits* in the *Clusters + outlier* condition committed after *Approach* and *Withdrawal* motivational cues, when compared to *Neutral*. That is, a relatively broad cognitive scope (associated to the *Neutral* cue), is expected to facilitate attentional and mnemonic allocation to the outlier word.

All three eye tracking variables were also expected to correlate with motivational cues. *Radius* was expected to be narrower after *Approach* and *Withdrawal* cues, when compared to *Neutral*. *Gaze duration* was expected to increase when motivational intensity was high (as more attention is paid to the “trees” rather than the “forest”). We also expected more words to be skipped after high motivation cues.

We also expected to find an interaction between *Cue* and *Layout* on *Errors*: a subset of visual arrangements may facilitate performance when they match the participants’ motivational state. Concentrated information might be less attended to when motivational intensity is low. The *Layout* factor was specified as nominal, and comprised five levels (one for each visual presentation layout).

We applied Bonferroni correction for 5 comparisons, corresponding to the total number of tests we performed.

#### Hypothesis 2: Physiology is influenced by motivational intensity and predicts errors

**2a**. We predicted a main effect of *Cue* on *FAA* (asymmetry), *IBI*, *ISCR*. More specifically, we expected *Approach* and *Withdrawal* to increase activation (decrease *IBI* and increase *ISCR*). Affects high in motivational intensity are expected to raise left hemispherical activity, increasing the *FAA* variable.

**2b**. We also investigated how physiology predicts performance. We tested the effect of *FAA*, *IBI*, *ISCR* and *Radius* on *Errors*. We assumed that since motivational intensity affects both performance and physiology, physiology should then predict performance. Because of the explorative nature of this test, we Bonferroni corrected all obtained p-values for 12 comparisons (4 variables × 3 studies).

#### Hypothesis 3: Affect modulates memory and interacts with search paradigm

We expected to observe a main effect of *Feedback* on *Errors*. The *Feedback* factor comprised five levels: *Neutral*, *Gain*, *Nongain*, *Loss*, *Nonloss*. Negative rewards (*Nongain*, *Loss*) were expected to increase the number of *Errors*, decreased instead by positive rewards (*Gain*, *Nonloss*). We also expected the magnitude of the effect to be higher for the most positive and most negative outcomes (e.g. *Loss* should increase errors more considerably than *Nongain*, see [75]).

A *Feedback* × *Layout* interaction on *Errors* was also predicted: negative rewards may reduce performance more evidently when information is displayed in sparse arrangements (e.g. random) whereas positive rewards may facilitate memory when utilising sparse visualisations.

### Results

All results obtained from Study 1 related to each hypothesis are listed in this section. Results from all studies will be summarised in the final section of the paper (Tables 1 to 3).

**Table 1:** Hypothesis 1: Summary of effect of motivational cues on errors, all studies.

	Study 1		Study 2		Study 3	
Factor	*p* ^‡^	*r_eq._*	*p* ^†^	*r_eq._*	*p* ^†^	*r_eq._*
*Approach*	**0.040**	.15	>0.2		>0.1	
*Withdrawal*	**0.005**	.22	**0.004**	.25	**0.032**	.40
Interaction*	>0.5		**0.016**	.80	>0.3	

* Study 1: *Cue* × *Layout*. Studies 2 and 3: *Cue* × *Diversity*.

^‡^ Study 1: p-values Bonferroni corrected for 5 comparisons.

^†^ Studies 2 and 3: p-values Bonferroni corrected for 4 comparisons.

**Table 2:** Hypothesis 2a: Summary of effect of cues on physiology, all studies (p-values Bonferroni corrected for 3 comparisons).

	Study 1		Study 2		Study 3	
Factor	*p*	*r_eq._*	*p*	*r_eq._*	*p*	*r_eq._*
***IBI***						
*Approach*	**<.001**	-.42	0.120		>0.2	
*Withdrawal*	**<.001**	-.27	>0.3		**0.024**	-.40
***ISCR***						
*Approach*	**<.001**	.37	>.500		**0.003**	.43
*Withdrawal*	**<.001**	.32	>.500		>0.1	
***FAA***	>.500		>.500		>.500	

**Table 3:** Hypothesis 2b: Predicting errors via physiology, all studies and metrics (Bonferroni corrected for 12 comparisons). In two studies (1 and 3) higher *Radius* was correlated to less errors. In Study 3, Increased arousal (shorter *IBI*) was correlated to less errors.

	Study 1		Study 2		Study 3	
Factor	*p*	*r_eq._*	*p*	*r_eq._*	*p*	*r_eq._*
*IBI*	>.500		>.500		**0.004**	.14
*ISCR*	>.500		>.500		0.070	
*Radius*	**<.001**	-.29	>.500		**0.006**	-.25
*FAA*	>.500		>.500		>.500	

#### Hypothesis 1: Motivational intensity modulates cognitive scope and interacts with search paradigm

Overall, the average number of errors committed per trial after *Neutral* cues (*M*: 3.15, *SD*: 1.51) was less than that committed after both motivational cues. Errors increased after both the *Approach* (*M*: 3.40, *SD*: 1.74), *t*(7185) = 2.67, *p* = .040, *r_equivalent_* = -.15, *N* = 40 and *Withdrawal* (*M*: 3.53, *SD*: 1.85), *t*(7185) = 3.20, *p* = .005, *r_equivalent_* = -.22, *N* = 40 cues.

Eye tracking data showed that the mean distance of the eye from the centre of the screen, in pixels, (*Radius*) was larger after the *Neutral* cue (*M*: 441.59, *SD*: 29.21), being shorter after the *Approach* cue (*M*: 403.51, *SD*: 69.91), *t*(5820) = -7.27, *p* < .001, *r_equivalent_* = -.41, *N* = 33 and the *Withdrawal* cue (*M*: 409.35, *SD*: 72.55), *t*(5820) = -7.16, *p* < .001, *r_equivalent_* = -.35, *N* = 33. Participants also skipped (did not read) a larger number of words per trial when compared to the *Neutral* cue (*M*: 1.70, *SD*: 0.61), skipping more after both the *Approach* cue (*M*: 2.16, *SD*: 1.32), *t*(2324) = 4.78, *p* < .001, *r_equivalent_* = .26, *N* = 33 and the *Withdrawal* cue (*M*: 2.12, *SD*: 1.36), *t*(2324) = 4.01, *p* < .001, *r_equivalent_* = .23, *N* = 33. *Gaze duration* (average time spent looking at every single word in one trial, in milliseconds) increased after the *Approach* cue (*M*: 520.06, *SD*: 263.27), *t*(18613) = 3.59, *p* = .001, *r_equivalent_* = .14, *N* = 33, when compared to the *Neutral* cue (*M*: 467.73, *SD*: 45.93).

We found a significant *Cue* × *Layout* interaction on gaze data, involving the *Scatter* and *List* layouts. In summary, *Scatter* was correlated to more reduced *Radius* after *Approach* (*M*: 416.57 pixels, *SD*: 71.03) when compared to *Neutral* (*M*: 451.10, *SD*: 33.15), *t*(5808) = 4.29, *p* < 0.001, *r_equivalent_* = -.38, *N* = 33. For *List*, *Radius* was slightly less constrained: (*M*: 195.14, *SD*: 34.90) was observed after *Approach* and (*M*: 205.06, *SD*: 29.94) *r_equivalent_* = -.26 after *Neutral*.

We found no significant effect of *Cue* on whether the outlier word within the *Clusters + outlier* condition was remembered (*p* = 0.110). It is also worth noting that we found no significant difference in overall performance between *Colours* and *Clusters* and no significant interaction between the *Cue* and *Layout* factors on *Errors*.

All tests were Bonferroni corrected for 5 comparisons, which corresponds to the total number of tests performed to assess this hypothesis.

#### Hypothesis 2: Physiology is influenced by motivational intensity and predicts errors

**2a**. *ISCR* and *IBI* were strongly correlated to participants’ motivational states elicited by the cues we employed. *ISCR* was higher, suggesting increased sympathetic nervous system activation after both the *Approach* (*M*: 65.17, *SD*: 55.51), *t*(6468) = 6.24, *p* < .001, *r_equivalent_* = .37, *N* = 36 and *Withdrawal* (*M*: 65.26, *SD*: 58.33), *t*(6466) = 2.18, *p* < .001, *r_equivalent_* = .32, *N* = 36 cues, when compared to *Neutral* (*M*: 55.28, *SD*: 44.91). *IBI* was shorter, again suggesting increased activation, after both the *Approach* cue (*M*: 794.17 ms, *SD*: 111.77), *t*(6467) = -6.22, *p* < .001, *r_equivalent_* = -.41, *N* = 36 and the *Withdrawal* cue (*M*: 797.40 ms, *SD*: 116.33), *t*(6467) = -4.93, *p* < .001, *r_equivalent_* = -.27, *N* = 36, when compared to the *Neutral* cue (*M*: 808.85 ms, *SD*: 114.42). All p-values were corrected for 3 comparisons. EEG alpha asymmetry (*FAA*) did not significantly correlate to motivational intensity in this study (*p* > .5).

**2b**. Exploratory prediction of errors using all 4 physiological variables (Bonferroni corrected for 12 comparisons, as described in the “Hypotheses” section in the introduction) was only significant for *Radius*. The highest half of *Radius* (representing broader attention) was associated to fewer errors (*M*: 3.27, *SD*: 1.46), *t*(5821) = -11.46, *p* < 0.001, *r_equivalent_* = .29, *N* = 33 than the lowest half: (*M*: 3.72, *SD*: 1.76).

#### Hypothesis 3: Affect modulates memory and interacts with search paradigm

Out of the four possible non-neutral rewards (*Gain*, *Loss*, *Nongain*, *Nonloss*), two (*Nongain* and *Nonloss*) were associated to an increased number of errors per trial. In detail, when compared to the *Neutral* reward (*M*: 3.15, *SD*: 1.51), more errors were committed after a *Nongain* reward (*M*: 3.55, *SD*: 1.73), *t*(7175) = 2.927, *p* = 0.003, *r_equivalent_* = .24, *N* = 40 and after a *Nonloss* reward (*M*: 3.42, *SD*: 1.84), *t*(7175) = 3.90, *p* < 0.001, *r_equivalent_* = .15, *N* = 40.

A significant *Feedback* × *Layout* interaction on number of errors per trial was found (*p* < .001). The interaction can be described as follows. In the baseline condition (*Scatter*), small increases of errors were associated to *Loss* rewards (*M*: 3.70, *SD*: 2.09) *r_equivalent_* = .10, *Gain* (*M*: 3.64, *SD*: 2.04) *r_equivalent_* = .08 rewards and *Nonloss* rewards (*M*: 4.07, *SD*: 1.90) *r_equivalent_* = .27, when compared to *Neutral* rewards (*M*: 3.47, *SD*: 1.58). The increase in errors due to *Loss* rewards was significantly greater for the *List* layout (*M*: 4.32, *SD*: 1.88), *t*(7175) = 2.44, *p* = 0.015, *r_equivalent_* = .34, *N* = 40 and the *Colours* layout (*M*: 3.18, *SD*: 1.94), *t*(7175) = 2.40, *p* = 0.016, *r_equivalent_* = .27, *N* = 40 (consider that the *Colours* layout was easier for participants, resulting in less average errors per trial after *Neutral* rewards (*M*: 2.60, *SD*: 1.59)). The *Colours* layout was also associated to a relative decrease in errors after *Gain* rewards were presented (*M*: 2.38, *SD*: 1.93), *t*(7175) = -1.99, *p* = 0.047, *r_equivalent_* = -.10, *N* = 40. The *Clusters + outlier* layout saw a limited increase in number of errors following *Nonloss* rewards (when compared to the *Scatter* layout) (*M*: 3.43, *SD*: 2.08), *t*(7175) = 2.44, *p* = 0.015, *r_equivalent_* = .03, *N* = 40.

#### Discussion

The first observation of this study is that motivational intensity, in both directions, narrowed visual attentional scope during the word search task. The effect of *Withdrawal* was stronger than that of *Approach*. This observation is supported by eye tracking data, which indicates that visual attentional scope narrowed as motivation increased: more attention was allocated to the central areas of the screen, gaze duration increased along with the number of words not read per trial. Hence, we attribute the increased number of errors after induction of high motivational intensity to visual narrowing of attention. No *Cue* × *Layout* interaction was found on *Errors*, unlike our prediction. However, a two-way interaction between *List* and *Scatter* was found on *Radius*, suggesting that the effect of motivational intensity on visual search varies depending on whether a list or a scatter is being displayed.

We interpret low *IBI* and high *ISCR* (measured to assess Hypothesis 2) as increases in activation (arousal). Given that arousal is expected to be particularly heightened by monetary rewards [80], this effect was not surprising. Contrary to our hypothesis, physiology (apart from eye tracking) did not directly predict errors. Nevertheless, the lack of a direct correlation between physiological variables related to activation and *Errors* suggests that reduced visual cognitive scope was mostly responsible for decreased performance. In other words, while arousal was elicited in expectation of the rewards, it did not directly influence performance or visual search strategy. The strong correlation between physiological variables and motivational intensity we found in our experiment indicates that it would be possible to infer arousal via ECG and EDA while performing visual searches. However, care must be taken in performing such reverse inferences; while motivational intensity can induce arousal, the reverse is not necessarily true. Combining arousal with eye tracking data into a single predictor would be more informative as all these metrics showed robust correlations to motivational intensity, given that *Radius* did predict errors. We attempted to further investigate these findings in the following two studies.

EEG asymmetry analysis did not yield significant results, preventing the prediction of motivational intensity from the *FAA* signal. This might be due to the difficulty of the task, as alpha activity is generally inversely correlated to brain activity, especially under high memory load [81]. This suggests that more computationally intensive analysis would be required to reveal correlations between EEG and motivational intensity when performing visual word searches.

We also observed an interaction between *Layout* and *Feedback*. Relatively more errors were committed after losses under the *List* and *Colours* conditions. This could be interpreted as a suppression of associative memory related to contextual information presented within the *Colours* and *List* layouts, induced by negative affect (*Loss*). Similarly, relatively less errors where committed in the *Colours* and *Clusters + outlier* condition after positive rewards (*Gain* and *Nonloss*. This suggests that that both positive and negative rewards affected memory, although the effect of negative rewards was more pronounced. These findings support dual-processing models in which negative affect (or stress) impairs hippocampal-dependent associative memory [78, 79]. That is, it has been previously indicated that negative affect at retrieval can impair associative memory without reducing memory for the task-relevant items themselves [77, 82]. This is particularly relevant for our context: in our case, we consider contextual information the colour of a word or its position within a list, which is not strictly task-relevant information. Consistently with this interpretation, our effect was also observed at retrieval since we presented reward feedback after the word search phase (which corresponded to the encoding phase).

In conclusion, Study 1 indicated that motivational intensity modulates attentional scope by altering visual search strategies. Among the five layouts we employed, we observed interactions between two of them: the *Scatter* and *List* conditions (on *Radius* and *Feedback*-induced errors). This suggests that these two styles of visualisation were differently affected by our manipulations. These two styles of visualisation are heavily used in computer systems, as mentioned in the introduction. Lists are ubiquitous, while scatters (such as tag clouds) have seen a rise in popularity in recent years [83]. Scattered information is also used in specialised visualisations for which lists would be ineffective (such as flight and naval control systems or star charts). Moreover, some systems display information using both a list and a scatter [29]. This led us to the development of the further two studies presented in the following sections, which focused on assessing whether motivational intensity and affect modulate visual search and physiology independently of visual presentation layout, and whether their effects are more pronounced when employing lists or scatters alone.

## Study 2

The previous study indicated that visual search and physiology are affected by motivational intensity, while affect (especially negative) influences memory at retrieval. It also indicated that visual search strategy, as measured by eye tracking variables, may not be equally altered by motivational intensity depending on whether information is presented within lists or scatters.

In this study, we investigated how motivational intensity affects visual search only when performed on lists, while the following study focused on scatters. Given that visual presentation layout is now fixed, we instead manipulated search paradigm by altering the amount of target categories. Participants were asked to identify words belonging to a number of target categories that varied from 1 to 3. We call this variable *Diversity* (information diversity).

Manipulation of *Diversity* is of interest to us since motivational intensity has been previously linked to conceptual scope [84]. Although, note that this research has been recently involved in a controversy [85], raising the importance of its replication. Moreover, working memory requirements have been shown to adversely affect visual search performance [86], suggesting that altering *Diversity* may differentially affect visual search strategy. In addition, working memory requirements may differentially affect lists or scatters. Manipulating *Diversity* has the added benefit of allowing assessment of linear effects in our hypothesis tests; that is, *Diversity* was treated as a numerical variable in our models in studies 2 and 3, whereas the *Layout* variable in Study 1 was nominal.

### Method

**Fig 3:**
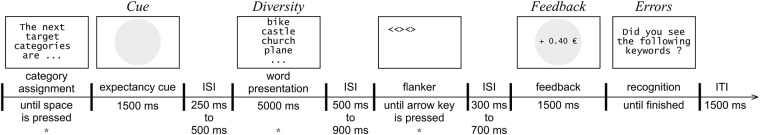
Trial structure, Study 2. The structure was similar to Study 1; asterisks mark the three phases in which the design differed from the first study. The differences were: 1) multiple categories were assigned 2) 12 words were presented, for a longer time, always using the same layout and 3) the flanker was displayed in a randomly selected quarter of the screen. This design was nearly identical to that employed in Study 3, which differed only in the arrangement of keywords. Above each phase, variable names of interest are indicated in italics.

The general trial structure was unchanged from Study 1. Three trial phases differed from Study 1: category assignment, word presentation and flanker. The three phases discussed below are marked with asterisks in Fig 3, which depicts this study’s trial structure.

#### Category assignment

During the category assignment phase, participants were instructed to identify words belonging to one, two or three target categories. The number of target categories was pseudorandomly selected and balanced across trials, within participants. The resulting design was a 3 × 3 × 3 (*Cue* × *Diversity* × *Feedback*) fractional factorial design, in which the *Cue* and *Diversity* factors were fully crossed. As in Study 1, we repeat each combination of the two fully crossed factors (*Cue* and *Diversity*) 12 times, resulting in 108 trials (3 × 3 × 12). The lower number of trials was compensated by longer trial length: words were presented for 5 s instead of 3 s.

We raised the number of categories from 12 to 14 for this study, by splitting the “animals” category into “birds” and “mammals” and by adding the “building tools” category (“työkalut” in Finnish). Since multiple categories could be displayed in a single trial, the only constraint in category selection was that the same target category could not be used twice in adjacent trials (in Study 1, the same category could not be used as neither target nor irrelevant in two adjacent trials).

#### Word presentation

Words were only presented in a list, uniformly distributed over the vertical axis of the monitor and centred horizontally. Word presentation duration was increased from 3 s to 5 s. We presented 12 words instead of 10 to allow at most 6 categories in total to be displayed with 2 words for each. This duration was determined after pilot testing, during which we aimed at an average recognition accuracy of 75% per participant.

An additional difference from Study 1 is that words were presented in random order, rather than alphabetical, to ease comparisons with Study 3.

#### Flanker task

In Study 1 we always presented the flanker at the centre of the screen, as done in previous experiments (e.g. [20]). A possible confound in this approach is that high motivation might provoke an anticipatory narrowing of attention towards the centre of the screen due to the flanker’s location. To reduce this possibility, in Studies 2 and 3 we presented the flanker in a randomly selected quarter of the screen. As with Study 1, we verified that participants were motivated by our manipulations by performing a one-tailed t-test on reaction times to flanker stimuli after the *Neutral* cue against the *Withdrawal* and *Approach* cues. Once again, the null hypothesis was rejected in all cases for both Studies 2 and 3.

#### Hypotheses

The high level interpretation of our hypotheses was kept unchanged from Study 1. In Studies 2 and 3, visual search paradigms were manipulated using an independent variable called *Diversity* (replacing *Layout*). *Diversity* was coded as a numeric variable.

- Hypothesis 1: motivational intensity modulates cognitive scope and interacts with search paradigm. We expected a main effect of *Cue* on *Errors* and eye tracking variables (*Radius*, *Gaze duration* and *Words skipped*). *Diversity* was a covariate in these tests, since a low number of categories can be more easily parsed and remembered. We also expected that a larger (vs. smaller) number of target categories would be more easily parsed and encoded into memory, especially when current attentional scope is broad (vs. narrow). Hence, we expected narrow-scope inducing cues (*Approach* and *Withdrawal*) to further increase the number of errors committed as *Diversity* increases, resulting in an interaction.
- Hypothesis 2: physiology is influenced by motivational intensity and predicts errors. This hypothesis was tested identically to Study 1. We assessed the effect of *Cue* on *FAA*, *IBI* and *ISCR* (Hypothesis 2a). We also explored the possibility of predicting *Errors* from *FAA*, *Radius*, *ISCR* and *IBI* (Hypothesis 2b).
- Hypothesis 3: affect modulates memory and interacts with search paradigm. Similarly to Study 1, we expected a main effect of *Feedback* on *Errors* and an interaction between *Feedback* and *Diversity* on *Errors*.

#### Participants

Twenty people (8 female) participated in the experiment. Their age ranged between 34 and 19 years (*M*: 26, *SD*: 4.69). The number of participants was decided after verifying that the results we obtained from Study 1 did not change, despite halving the number of participants. Data from one of these participants (a 25 years old male) were discarded as the participant noticed that rewards were not being awarded fairly.

### Results

Study 2 results are displayed below, ordered by hypothesis. Results from all studies are summarised in the final section of the paper and in Tables 1 to 3.

#### Hypothesis 1: Motivational intensity modulates cognitive scope and interacts with search paradigm

A significant effect of *Withdrawal* cues was found, so that participants committed more errors (*M*: 7.14, *SD*: 1.61), *t*(2046) = 3.23, *p* = 0.004, *r_equivalent_* = .25, *N* = 18 when compared to *Neutral* cues (*M*: 6.88, *SD*: 1.46).

Cues significantly interacted with diversity. Participants committed a higher number of errors as diversity increased, after *Neutral* cues. This increase was due to the higher difficulty of remembering more categories. However, the increase in errors was less pronounced after *Withdrawal* cues. In detail, the lowest number of errors was found in the pair of *Neutral* cue and one target category (*M*: 5.18, *SD*: 1.60). The errors where highest in *Neutral* and three target categories than all other combinations (*M*: 8.62, *SD*: 1.32). This resulted in a significant interaction with *Withdrawal*, (*M*: 8.41, *SD*: 1.43), *t*(2046) = -2.87, *p* = 0.016, *r_equivalent_* = .80, *N* = 18.

Eye tracking data showed a reduced distance of gaze from the centre of the screen (*Radius*) after the *Approach* (*M*: 243 pixels, *SD*: 53), *t*(1776) = -4.33, *p* < 0.001, *r_equivalent_* = -.29, *N* = 17 and *Withdrawal* (*M*: 243 pixels, *SD*: 50), *t*(1776) = -4.02, *p* < 0.001, *r_equivalent_* = -.33, *N* = 17 cues, when compared to *Neutral* (*M*: 251 pixels, *SD*: 44). The remaining eye tracking variables, *Words skipped* and *gaze duration*, were not significant.

All p-values were Bonferroni corrected for 4 comparisons.

#### Hypothesis 2: Physiology is influenced by motivational intensity and predicts errors

**2a**. None of the physiological variables we employed (*FAA*, *ISCR* and *IBI*) correlated with *Cue* in this study (after correcting p-values for 3 comparisons).

**2b**. Prediction of errors could not be carried out using any physiological variable (*p* > .5), Bonferroni corrected for 12 comparisons.

#### Hypothesis 3: Affect modulates memory and interacts with search paradigm

A main effect of reward was found: *Loss* rewards induced more errors (*M*: 7.41, *SD*: 1.50), *t*(2042) = 3.64, *p* = 0.009, *r_equivalent_* = .48, *N* = 18 than *Neutral* rewards (*M*: 6.89, *SD*: 1.45).

A significant *Diversity* × *Feedback* interaction was found *t*(2042) = -2.86*p* = 0.004, indicating that while the *Loss* reward was correlated to more errors, this increase was gradually reduced as the number of target categories increased. More in detail, when considering one target category, *Loss* (*M*: 6.29, *SD*: 1.86) saw a large increase in comparison to *Neutral* (*M*: 5.18, *SD*: 1.60) *r_equivalent_* = .63. In contrast, with two target categories there was a smaller increase in errors, from (*M*: 6.85, *SD*: 1.99) to (*M*: 7.25, *SD*: 1.65) *r_equivalent_* = .22. With three target categories, *Loss* (*M*: 8.67, *SD*: 1.72) we saw a negligible decrease in errors when compared to *Neutral* (*M*: 8.62, *SD*: 1.32) *r_equivalent_* = .02.

### Discussion

We found a significant interaction between search paradigm and motivation (*Cue* and *Diversity*). However, the found interaction was the reverse of what we expected. While we predicted that narrowing of attention caused by high motivational intensity would further increase number of errors, the number of errors increased more steeply after *Neutral* cues, especially when compared to *Withdrawal* cues (marginally after *Approach* cues).

The effect of motivational intensity on gaze variables was more limited when compared with Study 1: only *Radius* was significantly affected by motivational intensity. Moreover, the main effect test of *Cue* on *ISCR* resulted not significant; it was suppressed by *Diversity* instead, suggesting that participants’ arousal increased in order to provide more resources when dealing with a more complicated task.

**Fig 4:**
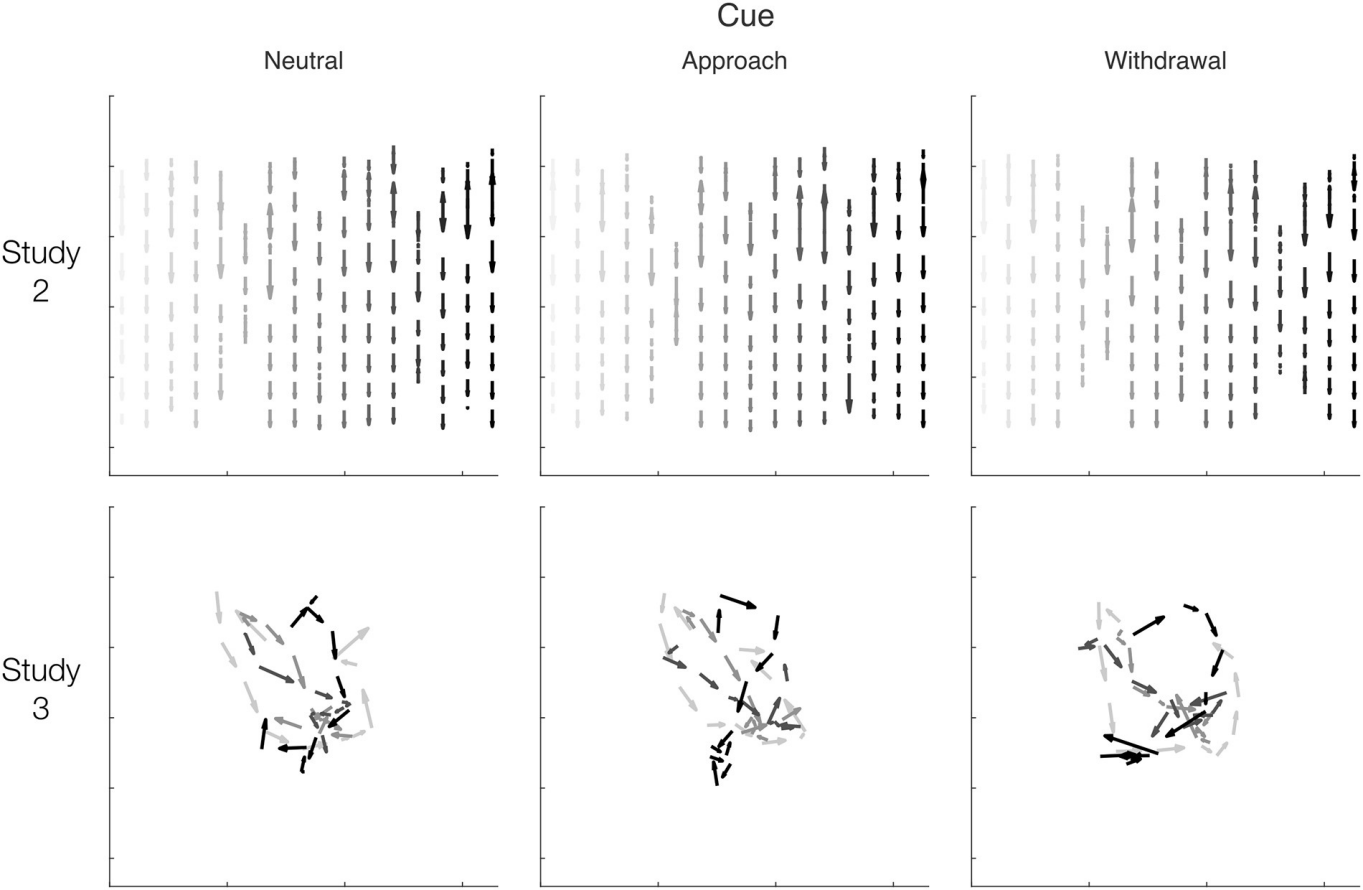
Gaze vectors, Studies 2 and 3. This figure shows the average gaze vectors for various participants, for each cue. Different shades of grey represent different participants. These were obtained by averaging the order in which words were gazed at in each trial. Note that direction and vertical spread for each participant appear to be relatively stable across cues in Study 2, when compared to Study 3. In the latter case, we can observe clear, seemingly random differences between different levels of *Cue*. This suggests that gaze patterns were less predictable in Study 3.

We believe these effects are due to the visual search strategy commonly employed when scanning lists. When words are presented in this format, most (if not all) participants would perform the search task by reading the list from top to bottom, word by word, until the allocated time expires. This would not be surprising: this approach is commonly employed in left to right languages such as Finnish. The presence of this strategy is supported by visual gaze vector inspection. We computed gaze vectors for each participant by averaging the order in which words were scanned (Fig 4). We argue that this relatively stable strategy reduces the impact of motivational intensity manipulations on visual attention. Following this premise, it is possible that the increased cognitive control induced by high motivational intensity [17], unable to subconsciously alter visual search strategy, instead facilitated memorisation. In fact, high motivation has previously been shown to increase working memory performance for information presented sequentially [87], a phenomenon which supports this explanation.

A static visual search strategy could also explain the *Diversity* × *Feedback* interaction. The most negative feedback (*Loss*) interacted with the number of target categories, having a larger impact when *Diversity* was low. This effect can have two possible non-dichotomic interpretations: firstly, it could be interpreted as a decrease in associative memory caused by losses (as in Study 1). As more words belonging to a single category are memorised sequentially, more associations are built into memory; these associations, however, can be disrupted by negative affect [77]. The second possibility is that the encoding phase was strengthened by increased resource allocation [17], as induced by the preceding motivational and *Diversity* cues. This would then counteract the disruption of associative memory caused by negative affect.

## Study 3

**Fig 5:**
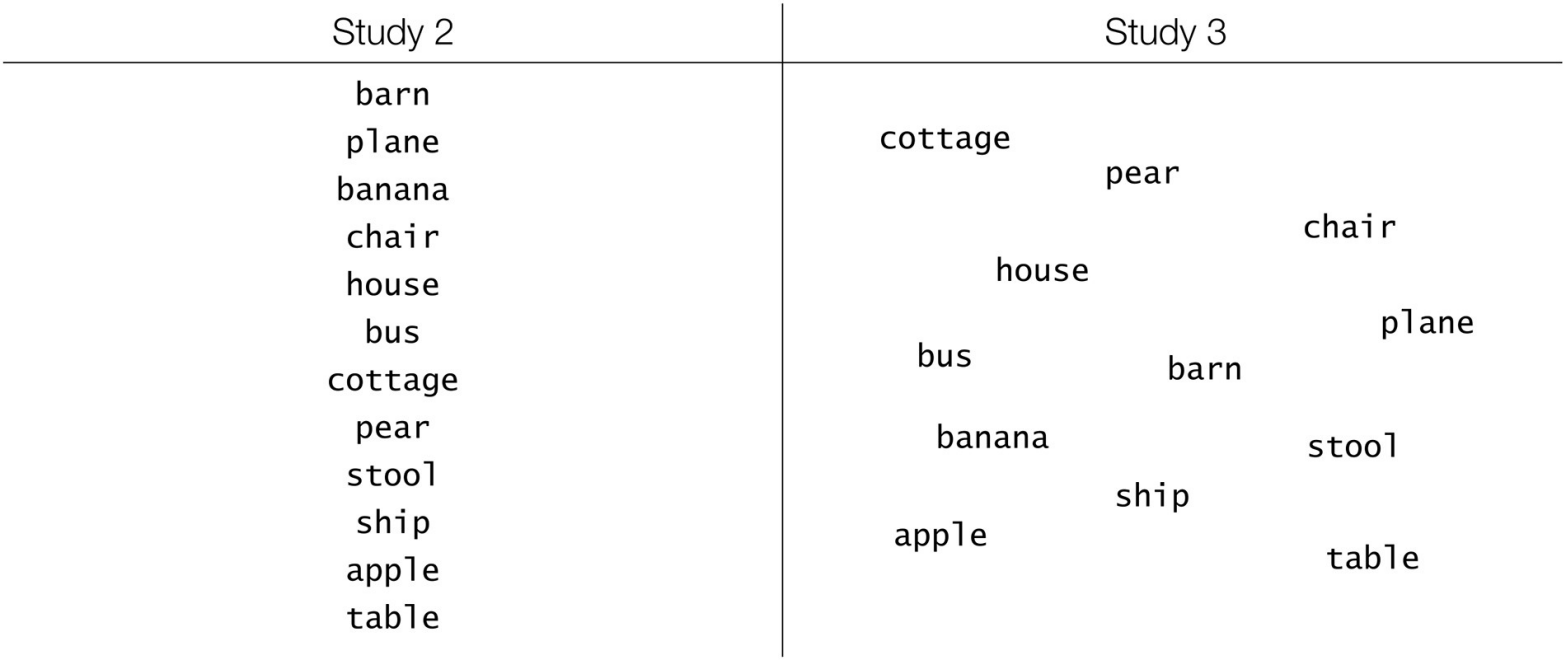
Word arrangements, Studies 2 and 3. This figure depicts example arrangements for the two studies. In both examples, 4 categories are present (fruits, vehicles, furniture and buildings). Since half of the categories were targets and half distractors, there were 2 target categories in this example (corresponding to a *Diversity* level of 2). Word arrangement was the only difference between Studies 2 and 3.

The only difference in design from Study 2 was the arrangement of keywords: in this study, we presented only pseudorandomly scattered words (corresponding to the *Scatter* layout in Study 1 and shown in Fig 5). Under all other aspects, this study was carried out using exactly the same parameters as Study 2.

### Participants

Twenty people (10 female) participated in the experiment. Their age ranged between 38 and 19 years (*M*: 25.15, *SD*: 4.57).

### Results

This section lists all results from Study 3, sorted by hypothesis. Results from all studies are summarised in the final section of the paper (Tables 1 to 3).

#### Hypothesis 1: Motivational intensity modulates cognitive scope and interacts with search paradigm

A main effect of cue was found for *Withdrawal* (*M*: 7.87, *SD*: 1.53), *t*(2152) = 2.66, *p* = 0.032, *r_equivalent_* = .40, *N* = 20, which induced more errors than *Neutral* (*M*: 7.35, *SD*: 1.29). Unlike Study 2, in this case we found no significant interaction between *Diversity* and *Cue* (*p* > 0.5).

Eye tracking data were consistently affected by motivational cues (especially when opposed to Study 2). *Radius* (mean distance of gaze from centre of screen), was narrower after both *Approach* (*M*: 426 pixels, *SD*: 65), *t*(1935) = -4.15, *p* < 0.001, *r_equivalent_* = .54, *N* = 18 and *Withdrawal* (*M*: 430 pixels, *SD*: 69), *t*(1935) = -3.07, *p* = 0.008, *r_equivalent_* = .52, *N* = 18 motivational cues (*Neutral*: (*M*: 462 pixels, *SD*: 38)). *Words skipped* (not gazed at, per trial) were more after both *Approach* (*M*: 1.93, *SD*: 1.41), *t*(1935) = 2.59, *p* = 0.038, *r_equivalent_* = .28, *N* = 18 and *Withdrawal* (*M*: 2.13, *SD*: 1.63), *t*(1935) = 4.48, *p* < 0.001, *r_equivalent_* = .37, *N* = 18 cues (*Neutral*: (*M*: 1.63, *SD*: 0.91)). *Gaze duration* was not significant for either *Approach* (marginally, *p* = 0.088) or *Withdrawal* (*p* ≥ 0.4).

All p-values were Bonferroni corrected for 4 comparisons.

#### Hypothesis 2: Physiology is influenced by motivational intensity and predicts errors

**2a**. Heart rate and skin conductance were correlated to motivational cues. Compared to *Neutral*, (*M*: 853 ms, *SD*: 163) *IBI* was significantly lower after *Withdrawal* cues (*M*: 840 ms, *SD*: 166), *t*(2043) = -2.65, *p* = 0.024, *r_equivalent_* = .40, *N* = 19 (it was not significantly lower after *Approach* cues, *p* > 0.2). *ISCR* was significantly higher after *Approach* (*M*: 53.40, *SD*: 36.01), *t*(2152) = 3.27, *p* = 0.003, *r_equivalent_* = .43, *N* = 20, *Neutral*: (*M*: 45.09, *SD*: 34.00). *FAA* did not yield significant results (*p* > 0.5). These p-values were Bonferroni corrected for 3 comparisons.

**2b**. Exploratory analysis for the prediction of errors (Bonferroni corrected for 12 comparisons) was significant for both *IBI* and *Radius*. The top half of trials which were associated to the highest IBIs (lower activation) was correlated to more errors (*M*: 7.61, *SD*: 1.89), *t*(2047) = 3.58, *p* = 0.004, *r_equivalent_* = .15, *N* = 19 when compared to the half related to lower *IBI* (higher activation) (*M*: 7.78, *SD*: 1.33). Similarly to Study 1, larger average distance of gaze from the centre (*Radius*) was associated to less errors (*M*: 7.56, *SD*: 1.19), *t*(1936) = -3.46, *p* = 0.006, *r_equivalent_* = -.25, *N* = 16 (lower *Radius*: (*M*: 7.91, *SD*: 1.45)). *ISCR* and *FAA* did not predict errors (*p* > 0.5).

#### Hypothesis 3: Affect modulates memory and interacts with search paradigm

A main effect of reward was found. Negative rewards were correlated to an increased number of errors, so that more were committed after *Nongain* cues (*M*: 7.75, *SD*: 1.19), *t*(2148) = 2.80, *p* = 0.005, *r_equivalent_* = .21, *N* = 20 and *Loss* cues (*M*: 7.96, *SD*: 1.55), *t*(2148) = 2.67, *p* = 0.007, *r_equivalent_* = .43, *N* = 20 (*Neutral*: (*M*: 7.35, *SD*: 1.29)).

Unlike Study 2, no *Diversity* × *Feedback* interaction was found *p* = 0.227.

### Discussion

In this study, we found a clear effect of *Cue* on visual attention, unlike Study 2. Among eye tracking variables, *Radius* and *Words skipped* were significantly affected by *Cue*. The amount of *Errors* was larger (effect size) for *Withdrawal* and significant for *Approach*. This suggests that the overall effect of motivational intensity on visual attentional scope is larger when words are presented in a scatter.

*Cue* affected both *IBI* and *ISCR*, unlike Study 2. *Withdrawal* significantly decreased *IBI*, while *Approach* (and marginally *Withdrawal*) were correlated to increased *ISCR*. The effect was less pronounced than that found in Study 1 (perhaps due to a lower number of participants). Nevertheless, this supports our hypothesis that high motivational intensity increases arousal during visual search, specifically by reducing *IBI* and increasing *ISCR*.

The comparison of gaze patterns between Study 2 and Study 3 (Fig 4) along with the effects previously discussed, leads us to the interpretation that scatters allows for greater flexibility in visual search strategy. The increased amount of liberty gave way to a greater suppression (narrowing) of visual attention, induced by high motivational intensity.

No interaction of *Feedback* and *Diversity* on *Errors* was found, unlike the other two studies. The absence of contextual information (which arises from sequential list parsing) in the visual arrangement of words in this study could explain why this was the case. A main effect of negative rewards, however, was present: *Loss* and *Nongain* both increased *Errors*. As predicted, the effect of *Loss* was greater than that of *Nongain*.

## Results summary

Results related to each hypothesis and additional analyses from all three studies are aggregated in this section.

### Hypothesis 1: Motivational intensity modulates cognitive scope and interacts with search paradigm

Effects on errors committed after reward expectation cues are summarised in Table 1. In general, high motivational intensity was correlated to an increased number of errors. The effect of *Withdrawal* was more pronounced than that of *Approach* (which was not significant in Study 2). A significant interaction was found in Study 2.

Gaze patterns were significantly correlated to both *Withdrawal* and *Approach* according to all three metrics (*Radius*, *gaze duration*, *Words skipped*) in Study 1. The outlier keyword in the *Clusters + outlier* condition was remembered and gazed upon less often after *Withdrawal* cues. In Study 2, a significant effect was found only for *Radius*. In Study 3, both *Words skipped* and *Radius* were significant.

In summary, high motivational intensity seemed to induce narrower visual attentional scope, as suggested by the number of errors committed and gaze patterns. This decrease was present but less pronounced in Study 2, for which we found only a main effect of *Withdrawal* and a *Cue* × *Diversity* interaction.

### Hypothesis 2: Physiology is influenced by motivational intensity and predicts errors

**2a**. Correlations between all physiological variables and motivational cues are listed in Table 2. Activation (as measured by *IBI* and *ISCR*) was generally increased by high motivational intensity. Once again, Study 2 presented different results than the other two studies: no effect of *Cue* on *ISCR* was found, but instead appeared to be suppressed by the effect of *Diversity*.

**2b**. Exploratory prediction of errors is summarised in Table 3. *Radius* appeared to be a good predictor for number of errors in all studies except Study 2. In Studies 1 and 3, reduced *Radius* was correlated to more errors, indicating that reduced visual scope decreased overall attention. Unexpectedly, in Study 3 increased arousal (shorter *IBI*) was correlated to *less* errors.

### Hypothesis 3: Affect modulates memory and interacts with search paradigm

Effects for all reward levels are displayed in Table 4. Negative rewards (*Nongain* and *Loss*) were associated to increased errors in all studies, *Nongain* being correlated to an increased number of errors in Study 1 and *Loss* in studies 2 and 3.

**Table 4:** Hypothesis 3: Summary of effect of reward feedback on errors, all studies.

	Study 1		Study 2		Study 3	
Factor	*p*	*r_eq._*	*p*	*r_eq._*	*p*	*r_eq._*
*Gain*	0.157		0.269		0.462	
*Nongain*	**0.003**	.24	0.119		**0.005**	.22
*Loss*	0.164		**<.001**	.48	**0.007**	.44
*Nonloss*	**<.001**	.15	0.105		0.093	
Interaction*	**0.002**		0.051		0.227	

* Study 1: *Feedback* × *Layout*; studies 2 and 3: *Feedback* × *Diversity*.

*Nongain* did not appear to significantly increase *Errors* in Study 2. Instead, we found a marginally significant but consistent interaction across the three levels of *Diversity* with *Loss*: the negative effect of *Loss* was gradually reduced as the number of target categories increased.

### Additional analyses

Results from the analyses introduced in the “Additional analyses” section in the Introduction are reported in this Section. These were not part our original hypothesis set. Instead, they were run to support our conclusions, as suggested by the reviewers of the present article.

#### Alpha power

The one-tailed, paired t-test comparing baseline alpha power against the middle second of the word search task was significant for 35/40 participants in Study 1, 19/20 participants in Study 2 and 15/20 participants in Study 3.

Alpha power was marginally correlated with *Diversity* in Study 2 (*p* = .06). This analysis did not yield significant results in Study 3 *p* > .50.

These results indicate that alpha band desynchronisation took place during the word search task phase, when compared to baseline. This result is consistent with previous work which indicated that reduced alpha band power is correlated with increased mnemonic and cognitive load [70–72].

The lack of significant results for the effect of *Diversity* on alpha band power in Studies 2 and 3 indicates that the difference in task difficulty caused by varying the number of categories was not great enough to be reflected by a detectable increase in alpha band power. That is, the number of words to be remembered did not vary (although the number of categories did), and this semantic difference was not sufficient to elicit a significant effect. It is also worth noting that the eyes were moving greatly during the word search task (especially in Study 3). Eye movement interference can explain the lack of significant results, particularly regarding Study 3, which displayed words scattered across the screen. Nevertheless, parietal alpha power was higher during baseline when compared to the word search task. This could explain the lack of significant findings for EEG asymmetry carried out to test Hypothesis 2.

#### Spill-over effect

We found a spill-over effect in Study 1: more errors when committed in the trials following *Nongain* (*M*: 3.53, *SD*: 1.77), *t*(7174) = 2.28, *p* = 0.045, *r_equivalent_* = .24, *N* = 40 and *Loss* (*M*: 3.51, *SD*: 1.67), *t*(7174) = 2.01, *p* = 0.004, *r_equivalent_* = .24, *N* = 40 when compared to trials that were preceded by *Neutral* (*M*: 3.30, *SD*: 1.55). No effects were found in Study 2 *p* = .41 and 3 *p* > .5. Given that our trials were randomised, it is unlikely that this spill-over effect confounded our main results. However, it is worth noting its presence, and is particularly interesting that only negative reward feedback cues elicited an effect. This is consistent with Hypothesis 3 results: negative rewards appeared to have a frequent detrimental effect on memory while the effect of positive rewards (in the opposite direction) was more rare and less strong.

#### Eye tracking calibration

We found no significant effect of trial number on the average distance between the closest word and gaze position in all studies (Study 1 *p* = .53, Study 2 *p* = .17, Study 3 *p* = .46). This indicates that calibration accuracy was relatively stable, given our experimental duration (approximately 1 hour and 30 minutes for Study 1 and 1 hour and 50 minutes for Studies 2 and 3).

## Overall discussion

Across the three studies, main effects of motivational intensity on gaze and performance were found in Study 1 and Study 3. In Study 1, an interaction on *Radius* (mean distance of gaze from centre) was found between *List* and *Scatter*. In Study 2, which employed a list, the main effect of motivational intensity was slightly reduced (being significant only for *Withdrawal*) while an interaction was found. Study 1 alone indicated that there was an effect of motivational intensity on visual search, which may differ depending on whether scatters or lists are displayed. Studies 2 and 3 indicated that the effect of motivational intensity on visual search patterns is more pronounced when scattered information is presented.

We interpret these results as an alteration of search strategies induced by high motivational intensity, which is more likely to be observed when scanning relatively sparse search spaces. When information is presented in a consistent format, such as the list used in Study 2, this effect is reduced. The interaction between motivational intensity and information density exclusively in Study 2 indicated that high motivational intensity slightly reduced the negative impact on performance normally caused by the memorisation of a large number of categories.

A possible explanation for these findings is that extrinsic monetary motivational manipulations acted as stressors, constraining visual attention. However, when information was parsed sequentially visual attention was less likely to be constrained. Cognitive resource allocation, in absence of any evident attentional constraint, allowed performance to be less affected by high levels of stress.

This indicates that the impact of motivational intensity varies greatly depending on the format in which the search task is organised. This is consistent with previous research which indicated that motivational intensity narrows visual cognitive scope [20]. Our observations are also consistent with previous research in which modulation of visual attention did not play a role in performance. It was observed that motivation increases memory by facilitating maintenance of information when rewards are presented before maintenance [87, 88]. Neurologically, this could be explained by increased activity in the lateral prefrontal cortex (LPFC) induced by motivation; it has previously been suggested that stimulating the LPFC can increase working memory capacity [89, 90]. Stimulating this area via motivational cues may then favour memory.

These effects, in our case, applied only to *Approach*-directed motivation. It is notable that *Withdrawal*-directed motivation reduced performance in all cases, including lists. It has been indicated that motivational direction stimulates different areas of the brain, explaining why Study 2 performance results were greatly affected by opposing motivational directions [91]. This further strengthens the idea that both directions of motivation should be considered when studying human behaviour.

It is also worth noting that in all studies the most positive reward outcome (*Gain*) was never found to increase performance under main effects tests. Negative rewards (*Loss* and *Nongain*) were instead associated to decreased performance, in all studies and the spill-over effect additional analysis. *Loss*, in particular, strongly reduced performance when participants were under heavier mental load (main effect on Studies 2 and 3). The effect size of losses was greater than that of nongains, as predicted. This supports previous work which indicated that the “default” cognitive scope is broad [20, 23]. Under this assumption, gains would not broaden scope. That is, cognitive scope would be mostly unchanged after positive rewards, preventing the observation of an increase in performance.

### Implications

Given that the attentional-narrowing effect of motivational intensity on lists was reduced, it appears that using vertical lists would be preferable to scatters—especially when users are expected to be exposed to extrinsic motivational stressors. This implies that UI elements such as tag clouds may be less effective than vertical lists, especially unsorted. Vertical lists have also been indicated as the preferred method of arranging words, as they facilitate visual search [65]. This observation applies especially to scenarios in which spatial location would not provide additional properties relatable to the given word; that is, lists should be preferred to scatters if a given word’s position within a two-dimensional space does not convey any relevant information. In any case, it is worth remembering that arranging words across a two-dimensional space is an approach vulnerable to attentional biases.

Human-computer interaction research which takes into consideration the affective state of users and their correlated behaviour, such as physiological computing [32] or affective computing [33] would benefit from the findings presented in this paper, which suggest that eye tracking data collected while scanning lists might not generalise to scattered information, and vice-versa. For example, designing a visual search task using lists might mask variability in visual attention across conditions, reducing the efficacy of eye tracking metrics.

In more general terms, theoretical research on motivation and affect should benefit from our findings, which indicated that these phenomena induce different behaviours depending on 1) the way information is presented and 2) whether approach or withdrawal-directed motivation is being elicited.

Prediction of errors from physiology indicated that *Radius* could be used as a relatively accurate predictor of performance when using sparsely organised information (being significant in Studies 1 and 3). Narrow radius was associated to more errors, supporting our hypothesis that narrowing of attention reduces performance. Such biases could be detected by eye tracking along with *IBI*, which significantly predicted errors in Study 3.

### Limitations

An important limitation of the present study is due to the use of monetary rewards. In this way, we only explored the effect of extrinsic motivation induced by tangible rewards; it is possible that motivation induced by non-tangible and / or intrinsic rewards may have produced different results. However, testing for intrinsic rewards would require a very different experimental design, as this type of reward would be very difficult to include in a trial-by-trial design.

Use of a visual flanker task may have also influenced some of our eye tracking variables or *Errors*, as attention may pre-emptively shift towards the centre of the screen in anticipation of the flanker. We attempted to limit this confound by using a blank of variable duration after the word search phase and altering the position of the flanker in Studies 2 and 3. Moreover, previous experiments on motivational intensity and visual attention reported similar results regardless of whether visual or less perceptual flanker tasks (such as linguistic tasks) were employed [92]. While there may be overall differences between the types of flankers tasks utilised, it is important to note that it is worth utilising all types of flanker, including the type we utilised. Moreover, potential effects induced by the flanker cannot account for all differences observed across conditions (such as gaze duration on individual words, or *ISCR*).

In Study 1 we employed only one visual layout that included an “outlier” (*Clusters + outlier*). Ideally, it would have been of interest to include a *Colours + outlier* condition, in which an irrelevant word would be coloured as target. This would have helped in further investigating whether the attentional narrowing of scope would also apply to sparse arrangements. However, our search tasks were quite demanding and the duration of Study 1 experimental sessions often exceeded 90 minutes (excluding set-up). Including an additional layout condition would have strained participants, considerably reducing the feasibility of the study.

It is possible that the *Diversity* variable in Studies 2 and 3 affected physiological arousal, as it could have been heightened in trials which asked participants to memorise a high number of categories. Conditions were, however, balanced within participants; all levels of *Diversity* were tested against all levels of *Cue*.

Unfortunately, EEG asymmetry tests were insignificant in all three studies. This could be attributed to the difficulty of the search task (as mentioned in our methodology, we calibrated trial length to obtain an average accuracy of 75%). As indicated by our additional analysis, alpha waves were suppressed (desynchronised) under high memory load when compared to baseline. It has also been previously suggested that asymmetry might only be observed during affective (emotional) manipulations of motivational intensity [91], although it has been observed under non-emotional paradigms as well [44]. Another possible reason for the null result might be provided by the short time of segments available for our analysis, given that sensitivity of time-frequency transforms is normally higher when using longer time segments [63]. It has also been suggested that lower alpha band may be more relevant for motivational manipulations [49]. We attempted separate post-hoc tests on lower and higher alpha (8–10 Hz and 10–12 Hz) but these also yielded non-significant results. It is also worth noting that our studies were aimed at identifying the most suitable physiological signal for motivational intensity estimation. From this point of view, our finding of *Radius* being more suitable than *FAA* for the purpose of real time estimation using a low number of sensors is valuable.

Pupillometry has been previously used to assess the effect of motivational intensity on cognitive control [93]. Pupillometry has also been used in recent visualisation studies to investigate the effect of confusion [94], and to predict learning curves [95]. Preliminary tests, however, suggested that this technique would have not been applicable to our set-up, as the pupil data we obtained was not sufficiently accurate.

### Future work

The strong eye tracking results we reported suggest that eye tracking alone might be suitable for a follow-up study on the effects of motivational intensity on eye movements during visual search. In this paper, we focused on lists and scatters as they were within the initial layout set of Study 1. However, future studies could focus on additional visual arrangements frequently utilised in computer systems, such as matrices or multiple vertical lists. More eye tracking features that we did not consider could then be utilised (such as distance between saccades) or features could be engineered depending on the type of visualisation utilised (e.g. dwell time on left / right portion of screen, if two vertical lists are presented). Moreover, we often found that the standard deviation for *Radius* was generally larger when motivational intensity was high. This suggests that there are additional variables that interact with motivational intensity, changing gaze behaviour. Investigating additional layouts with more eye tracking variables, and potentially different types of flanker tasks (such as auditory) could help in identifying which factors are most closely correlated to changes in visual search patterns elicited by motivational intensity, and which gaze-related features are most suitable for motivational state estimation.

Interestingly, in Study 3, lower *IBI* was associated to *less* errors. This may indicate that higher sympathetic nervous system activation was elicited by motivational intensity, and increased performance during trials in which participants’ visual attention was not concurrently narrowed because of a latent variable (or noise). Heart rate (and hence IBI) increase is associated to stronger mental requirements [96], which were relatively higher in Studies 2 and 3. Further research would be needed to disentangle the relationship between withdrawal-directed motivation, *IBI* and performance during cognitively demanding word search.

Regulatory focus theory [97] indicates that approach-oriented individuals might benefit from approach-related motivation. This phenomenon was not tested in our experiment as our main aim was to assess general effects of motivational manipulations on visual scope. Exploring trait effects would require a larger, more balanced (in terms of traits) set of studies.

Future experiments inspired by our design would benefit from the inclusion of recall tests, apart from recognition tests. This is because high motivation induced participants in spending more time reading individual words, as measured by our *Gaze duration* variable. This would be useful in investigating whether high motivation increased encoding for individual words at the expense of recall rate for the remaining words—i.e. whether the “trees”, rather than the “forest”, were easier to remember. This recall-recognition tradeoff could be used to improve current models of visual search to include the additional dimension of motivational intensity [98].

## Conclusion

The presented studies indicated that motivational intensity affects visual search strategies, especially when information is presented in sparse layouts. When information was presented in lists, this effect was reduced, being absent for approach-directed motivation. We concluded that increased cognitive control induced by high approach-directed motivation increases memory during encoding when visual attention is not constrained, as lists are normally parsed sequentially.

When scanning sparse layouts, our findings indicate that eye tracking metrics would be beneficial in detecting attentional biases. Negative affect (*Loss*) has been shown to greatly reduce performance when performing visual word search under heavy mental load.

## Supporting information

S1 File This file contains six data tables, two tables per study and a “readme”. For each study, one table contains trial-level data while the other contains word-level data. These tables were used to compute our statistics, as outlined in the “Statistical analysis” section. The ‘README’ file contained within the zip file explains the contents of the table files, which are in csv format.
(ZIP)
